# Expression and Prognostic Significance of PD-L2 in Diffuse Large B-Cell Lymphoma

**DOI:** 10.3389/fonc.2021.664032

**Published:** 2021-06-10

**Authors:** Qianhui Gu, Jing Li, Zhuolin Chen, Jie Zhang, Hui Shen, Xiaobing Miao, Ying Zhou, Xiaohong Xu, Song He

**Affiliations:** ^1^ Department of Pathology, Affiliated Tumor Hospital of Nantong University, Nantong, China; ^2^ Department of Oncology, Affiliated Tumor Hospital of Nantong University, Nantong, China; ^3^ Cancer Research Center, Affiliated Tumor Hospital of Nantong University, Nantong, China

**Keywords:** diffuse large B-cell lymphoma, programmed death ligand-2, overall survival, progression-free survival, immunohistochemistry

## Abstract

Recent studies suggest that programmed death ligand-2 (PD-L2) constitutes an important antitumor immune response. Here, we investigated the relationship between PD-L2 expression and clinicopathological features in diffuse large B-cell lymphoma (DLBCL). Immunohistochemistry showed that positive expression of PD-L2 was observed in 45 of 181 newly diagnosed patients, including 14 cases with expression exclusively on tumor cells (TCs) and 31 cases with the expression on both TCs and immune cells (ICs) in the tumor microenvironment (TME). In 21 recurrent patients, positive expression of PD-L2 was present in six cases, including two cases with expression exclusively on TCs, and four cases with the expression on both TCs and ICs in the TME. Patients with PD-L2 tumor proportion score (TPS) ≥1% exhibited a better ECOG performance status (PS) (ECOG PS score <2, *P* = 0.041), lower international prognostic index (IPI) score (*P* < 0.001), and early Ann Arbor stage (Ann Arbor stage I or II, *P* = 0.010). Similarly, patients with PD-L2 immune proportion score (IPS) ≥1% also exhibited a better ECOG PS (ECOG PS score < 2, *P* = 0.006) and lower IPI score (*P* = 0.001). Survival analysis showed that patients with PD-L2 TPS ≥1% exhibited prolonged overall survival (OS) and progression-free survival (PFS). However, survival analysis showed no prognostic significance based on expression of PD-L2 on ICs in the TME. TC PD-L2 expression was significantly associated with OS (*P* = 0.041) and PFS (*P* = 0.001). In the multivariate analysis, TC PD-L2 expression was an independent prognostic risk factor for PFS (*P* = 0.013), but not for OS (*P* = 0.249). Furthermore, we found that higher TC and IC PD-L2 expression was associated with higher objective response rate (ORR). Moreover, we demonstrated that the expression level of PD-L2 was positively correlated with the expression status of M1 macrophage markers CD86. Our findings highlight PD-L2 as a promising therapeutic target in DLBCL.

## Introduction

Diffuse large B-cell lymphoma (DLBCL) is an aggressive lymphoma with a wide range of clinical manifestations, which accounts for almost 30% of all non-Hodgkin lymphoma cases (1). The therapeutic effects and prognosis of DLBCL patients have remarkably improved since the widespread applications of rituximab (2, 3). Despite standard therapy, ultimately 30 to 40% of DLBCL patients do not experience satisfactory outcomes and die from disease recurrence and progression (4). Therefore, it is necessary to identify these patients as early as possible and to prevent tumor progression and prolong survival time through relevant treatment measures.

Over the last few decades, significant advances in targeted immunotherapy have been made (5, 6). Based on the mechanism of tumor immune escape mediated by the programmed cell death 1 (PD-1) axis, PD-1/programmed cell death ligand 1 (PD-L1) inhibitors have shown clinical efficacy alone or in combination with traditional therapies. Unfortunately, PD-1/PD-L1 inhibitors have not been used in the clinical treatment of DLBCL patients. As the study progressed, studies have found that the expression status of PD-1, PD-L1 as well as Programmed death ligand 2 (PD-L2) also affects the clinical efficacy of conventional treatments such as surgery and chemotherapy (7, 8). Thus, tumor immune escape caused by PD-1 axis may also affect the survival time of tumor patients under the inhibitor treatment strategy of non-immune checkpoints.

PD-L2, as another ligand of PD-1, which binds to PD-1 regulating immune responses inducing immunological tolerance, its expression status is related to the clinical efficacy of immune checkpoint blockers targeting PD-1/PD-L1 (9, 10). However, some studies have shown that when PD-L2 is expressed in dendritic cells, it plays a stimulating role in T cell response and T cell proliferation rather than inhibition (11). Unlike PD-L1, PD-L2 has its own unique expression pattern (12). Furthermore, PD-L2 has an increased affinity for PD-1 (13), PD-L2 binds to PD-1 and produces different biological effects depending on its own antigen level (14). In addition, it has been found that PD-L2 is also involved in intracellular signaling pathways to promote tumor cell migration, invasion, and induce drug resistance (15, 16). It can be seen that PD-L2 plays an important role in the development and progression of malignant tumors.

In solid tumors, studies have shown that high expression of PD-L2 is associated with shorter disease-free survival (DFS) and overall survival (OS) in lung adenocarcinoma (17), esophageal cancer (18), gastric cancer (19), and hepatocellular carcinoma (20). Among renal cell carcinoma patients, the high expression of PD-L2 indicates progression-free survival (PFS) shortening (17). However, some studies hold different views; Shinchi et al. (21) and Matsubara et al. (22) found that lung cancer patients with high PD-L2 expression had longer DFS and PFS than those with low PD-L2 expression. In hematological tumors, PD-L2 can reflect the degree of immune cell infiltration in follicular lymphoma (FL) and low density of PD-L2 was the most sensitive/specific marker to segregate patients with adverse outcomes (23). These findings reflect that the expression status of PD-L2 may still affect the clinical efficacy in the absence of PD-1/PD-L1 checkpoint inhibitors, and the prognostic significance of PD-L2 in malignant tumors is still controversial. Its expression in different cells may play different roles and result in a different prognosis.

However, studies on the expression of PD-L2 in DLBCL and its prognostic significance are limited, and the results of PD-L2 expression rate are inconsistent (24–26). In this study, we used immunohistochemistry to analyze the expression of PD-L2 on tumor cells (TCs) and immune cells (ICs) in the tumor microenvironment (TME) in DLBCL.

## Materials and Methods

### Clinical Samples

This study included 198 DLBCL patients, 90 males and 108 females, with a median age of 66 years, who were diagnosed at the Affiliated Tumor Hospital of Nantong University from 2013 to 2018. Because four of them had both primary and recurrent tissues, 202 paraffin sections were used for immunohistochemistry, 181 primary tissues and 21 recurrent tissues. The deadline for survival follow-up was December 31, 2019, and the clinicopathological information involved was obtained from the medical record system. This experiment was approved by the Medical Ethics Committee of Nantong Cancer Hospital, Jiangsu Province, and informed consent was obtained from patients.

### Immunohistochemistry and Evaluation Criteria

Paraffin sections were heated at 80°C for 10 min, dewaxed in xylene, dehydrated by gradient alcohol, antigen retrieval was performed in EDTA solution for 2.5 min, 0.3% hydrogen peroxide for blocking endogenous peroxidase, and PD-L2 (clone D7U8C, Cell Signaling, USA, 1:100) was incubated overnight as a primary antibody. Secondary antibodies were incubated for 1 h, detected using 3, 3-diaminobenzidine tetrahydrochloride (DAB), counterstained with hematoxylin, and sealed with neutral gum. Immunohistochemical staining was independently assessed by two pathologists who were unaware of the patient’s diagnosis and clinical information. For PD-L2, according to tumor proportion score (TPS) and immune proportion score (IPS), membrane and cytoplasm staining were considered positive expression, divided into three grades: 0, negative: TPS/IPS <1%; 1, weakly positive: TPS/IPS: 1–49%; 2, strongly positive: TPS/IPS ≥50%.

### GEO Data Source

Two cohorts of DLBCL patients, GSE32918 and GSE10846, were obtained from the Gene Expression Omnibus (GEO) (https://www.ncbi.nlm.nih.gov/geo/) database to assess the relationship between the level of PD-L2 mRNA and prognosis. The data set met the following criteria: (1) PD-L2 mRNA level was detected; (2) Tissue samples; (3) Chemotherapy regimen is R-CHOP regimen or CHOP regimen; (4) Follow-up information includes survival time and survival status.

### Multiple Fluorescence Staining

The multiplex fluorescence staining kit was purchased from Absin kit (item number: abs50012). The steps were as follows: paraffin sections were heated at 80°C for 10 min and dewaxed in xylene, gradient alcohol dehydration, 10% neutral formalin immersion for 10 min; antigen was repaired in EDTA solution using microwave repair and cooled to room temperature, after 10 min of blocking, PD-L2 (clone D7U8C, Cell Signaling, USA, 1:100) was incubated overnight as an antibody; the secondary antibody was incubated for 10 min then incubated with fluorescent dye for 10 min, After the microwave repair was cooled to room temperature, the above steps were repeated again to complete the staining of CD68 (clone PG-M1, DAKO, Carpinteria, CA, 1:500), CD86 (clone E2G8P, Cell Signaling, USA, 1:150), and finally incubated with DAPI for 5 min to stain the nucleus and anti-fluorescence quencher seal. The expression was observed under fluorescence microscope.

Evaluation criteria of multiplex immunofluorescence staining: membrane and cytoplasm staining was considered positive expression, and the expression rate of PD-L2 still refers to TPS. CD68 staining was used to distinguish macrophages, the expression rate of macrophages PD-L2 was the percentage of PD-L2 and CD68 double positive cells in CD68 positive cells. Simultaneous staining of CD86 was used to distinguish M1 macrophages, the expression rate of M1 macrophages was the percentage of CD86 positive cells in total cells.

### Immune Cell Infiltration Analysis

A deconvolution algorithm based on CIBERSORT gene expression (http://cibersort.stanford.edu/) (27) was used to evaluate the immune cell infiltration in 29 DLBCL patients obtained from the TCGA database (https://portal.gdc.cancer.gov). The samples were divided into two groups according to the average expression of PD-L2. Wilcoxon Signed Rank test was used to compare immune cell content between groups with high and low expression of PD-L2 mRNA. Heatmaps and violin plots were analyzed and plotted using the packages “pheatmap” and “vioplot”, respectively.

### Statistical Analysis

The above data statistics were performed using SPSS 25.0 software and GraphPad Prism 8. Chi square test was used to compare the clinicopathological characteristics of TC/IC PD-L2 positive and negative patients and the clinical efficacy of the six cycle CHOP regimen. Fisher’s exact test was used to compare clinical outcomes between TC/IC PD-L2-positive and -negative patients who received six cycles of R-CHOP regimen. PFS was calculated from the day of diagnosis to the day of progression, relapse, or death. OS was calculated from the day of diagnosis to the day of death by any cause. Survival analysis was performed by log-rank test. Univariate and multivariate analyses were conducted using Cox proportional hazards regression model, and Omnibus test was used to verify the reliability of the model. The correlation between TC PD-L2 and M1 macrophage specific marker expression was tested by Pearson test. The difference was statistically significant at *P < *0.05.

## Results

### Expression of PD-L2 on TCs and ICs in the TME

**Figure 1:**
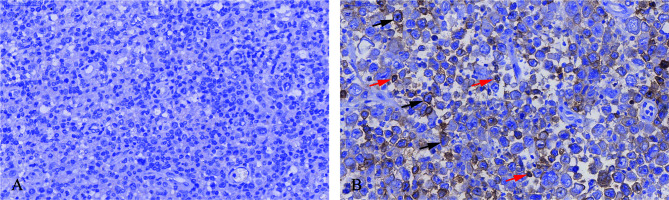
Immunohistochemistry staining of PD-L2 in DLBCL. **(A)** PD-L2 negative staining (magnification ×40). **(B)** PD-L2 positive staining (magnification **×**40). In **(B)**, arrows indicate PD-L2 expression on TCs (black arrows) and ICs in the TME (red arrows).

Immunohistochemical staining was performed in paraffin-embedded sections from 202 DLBCL patients. Most DLBCL patient tissues were negative for PD-L2 staining (**Figure 1A**). Among the positive tissues, PD-L2 was found in the membrane and in cytoplasm (**Figure 1B**). In 181 newly diagnosed patients, positive expression of PD-L2 was observed in 45 cases, including 14 cases with expression exclusively on TCs and 31 cases with the expression on both TCs and ICs in the TME (**Table 1-1**). In 21 recurrent patients, positive expression of PD-L2 was present in six cases, including two cases with expression exclusively on TCs, and four cases with the expression on both TCs and ICs in the TME (**Table 1-2**). Of the 181 newly diagnosed cases, positive expression of PD-L2 on TCs was higher than that on ICs in the TME. Intriguingly, positive expression of PD-L2 on TCs and ICs in the TME in recurrent cases was slightly higher than that in newly diagnosed patients.

**Table 1-1:** The number and positive rate of tumor cell PD-L2 in newly diagnosed and recurrent diffuse large B-cell lymphoma patients.

	Tumor cell	
	newly diagnosed patients	relapsed patients
TPS < 1%	136	15
TPS 1–49%	35	5
TPS ≥ 50%	10	1
Positive rate(TPS ≥ 1%)	24.86%	28.57%

**Table 1-2:** The number and positive rate of tumor microenvironment PD-L2 in newly diagnosed and recurrent diffuse large B-cell lymphoma patients.

	Tumor microenvironment	
	newly diagnosed patients	relapsed patients
IPS < 1%	150	17
IPS 1–49%	27	3
IPS ≥ 50%	4	1
Positive rate(IPS ≥ 1%)	17.13%	19.05%

### Relationship Between Expression of PD-L2 and Clinicopathological Characteristics

The relationship between PD-L2 and clinicopathological parameters is shown in **Table 2**. Among 181 newly diagnosed patients, patients with PD-L2 TPS ≥1% exhibited a better ECOG performance status (PS) (ECOG PS score < 2, *P* = 0.041), lower international prognostic index (IPI) score (*P* < 0.001), and early Ann Arbor stage (Ann Arbor stage I or II, *P* = 0.010). Similarly, patients with PD-L2 IPS ≥1% also exhibited a better ECOG PS (ECOG PS score < 2, *P* = 0.006) and lower IPI score (*P* = 0.001). In addition, positive expression of PD-L2 on TCs and ICs in the TME in EBER (Epstein–Barr virus-encoded small RNA)-positive cases was higher than that in EBER-negative patients. Due to lack of sufficient samples, correlation analysis between PD-L2 expression status and clinicopathological parameters in recurrent patients was not performed.

**Table 2:** Relationship between clinicopathological features and PD-L2 expression in newly diagnosed DLBCL patients.

Clinicopathological parameters	n	Tumor cells		P value	Immune cells in the TME		P value
		negative	positive		negative	positive	
Gender				0.824			0.833
Male	79	60	19		66	13	
Female	102	76	26		84	18	
Age(year)				0.620			0.268
<60 years	55	40	15		43	12	
≥60 years	126	96	30		107	19	
Extra nodal invasion				0.136			0.198
<2	116	83	33		93	23	
≥2	65	53	12		57	8	
Tumor size				0.503			0.517
<10 cm	152	116	36		126	26	
≥10 cm	18	15	3		16	2	
Hans classification				0.932			0.513
Non-GCB	144	108	36		118	26	
GCB	37	28	9		32	5	
ECOG PS				0.041			0.006
<2 score	37	23	14		25	12	
≥2 score	144	113	31		125	19	
B symptom				0.512			0.804
Absent	160	119	41		133	27	
Present	21	17	4		17	4	
Ann Arbor stage				0.010			0.051
I + II	71	46	25		54	17	
III + IV	110	90	20		96	14	
IPI score				<0.001			0.001
0–2	55	30	25		38	17	
3–5	126	106	20		112	14	
LDH				0.104			0.480
<211 U/L	44	29	15		38	6	
≥211 U/L	137	107	30		112	25	
*β*2-MGG				0.737			0.676
<4 mg/L	58	46	12		49	9	
≥4 mg/L	25	19	6		22	3	
ALB				0.173			0.513
<35 g/L	37	31	6		32	5	
≥35 g/L	144	105	39		118	26
EBER				0.041			0.019
negative	142	107	35		118	24	
positive	14	7	7		8	6	
Bcl-6				0.648			0.221
negative	24	19	5		22	2	
positive	135	101	34		110	25	
C-myc				0.928			0.903
negative	18	13	5		14	4	
positive	86	63	23		68	18	
Bcl-2				0.313			0.170
negative	28	19	9		20	8	
positive	84	65	19		70	14	
CD20				0.993			0.722
negative	8	6	2		7	1	
positive	173	130	43		143	30	

### Prognostic Significance of PD-L2 Expression

**Figure 2:**
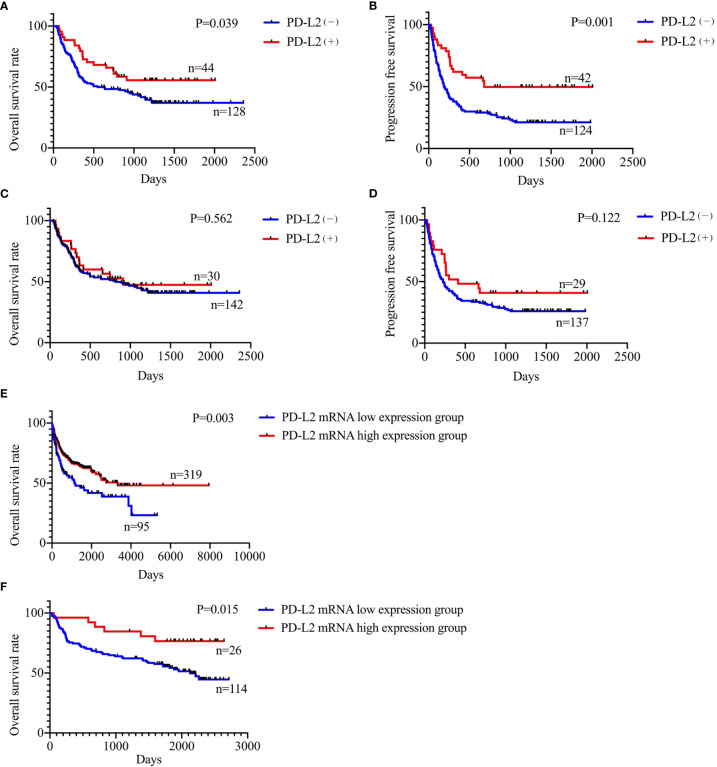
Relationship between TC PD-L2 (+) and overall survival **(A)** and progression free survival **(B)** curves. Relationship between IC PD-L2 (+) and overall survival **(C)** and progression free survival **(D)** curves in newly diagnosed diffuse large B-cell lymphoma. Relationship between PD-L2 mRNA level and overall survival of DLBCL. **(E)** GSE10846, **(F)** GSE32918.

Of the 181 newly diagnosed patients, 24 cases were lost to follow-up. Therefore, the 24 patients were excluded from the prognosis analysis. Survival analysis showed that patients with PD-L2 TPS ≥1% exhibited prolonged OS and PFS (**Figures 2A, B** **)**. However, survival analysis showed no prognostic significance based on expression of PD-L2 on ICs in the TME (**Figures 2C, D** **)**. Subsequently bioinformatics analysis based on GEO database showed that patients with high PDCD1LG2 mRNA level had a trend towards improved OS (**Figures 2E, F** **)**. The univariate analysis demonstrated that TC PD-L2 expression was significantly associated with OS (*P* = 0.041) and PFS (*P* = 0.001). In the multivariate analysis, TC PD-L2 expression was an independent prognostic risk factor for PFS (*P* = 0.013) (**Table 3**), but not for OS (*P > 0.05*) (**Table 4**).

**Table 3:** Univariate and multivariate analyses of clinicopathological factors associated with overall survival in DLBCL patients.

	Univariate analysis		Multivariate analysis	
	HR(95%CI)	P value	HR(95%CI)	P value
Age	2.279 (1.391–3.736)	0.001	–	–
Extra nodal invasion	1.695 (1.125–2.554)	0.012	–	–
ECOG PS	2.494 (1.361–4.571)	0.003	–	–
Ann Arbor stage	2.358 (1.516–3.668)	<0.001	–	–
LDH	2.217 (1.311–3.749)	0.003	–	–
IPI score	3.259 (1.926–5.514)	<0.001	0.452 (0.258–0.794)	0.006
B symptom	5.006 (2.957–8.476)	<0.001	0.290 (0.169–0.496)	<0.001
ALB	0.283 (0.179–0.447)	<0.001	2.396 (1.488–3.859)	<0.001
PD-L2(TPS ≥ 1%)	0.592 (0.358–0.978)	0.041	0.739 (0.442–1.236)	0.249

**Table 4:** Univariate and multivariate analyses of clinicopathological factors associated with progression free survival in DLBCL patients.

	Univariate analysis		Multivariate analysis	
	HR (95% CI)	*P* value	HR (95% CI)	*P* value
Age	1.795 (1.189–2.711)	0.005	–	–
Extra nodal invasion	1.636 (1.132–2.365)	0.009	–	–
ECOG PS	1.830 (1.107–3.026)	0.018	–	–
Ann Arbor stage	2.096 (1.409–3.119)	<0.001	–	–
LDH	2.097 (1.293–3.401)	0.003	–	–
IPI score	2.632 (1.665–4.162)	<0.001	0.568 (0.347–0.930)	0.024
B symptom	2.945 (1.793–4.838)	<0.001	0.466 (0.280–0.775)	0.003
ALB	0.342 (0.226–0.518)	<0.001	2.192 (1.422–3.380)	<0.001
PD-L2(TPS ≥ 1%)	0.447 (0.278–0.719)	0.001	0.540 (0.332–0.879)	0.013

### Relationship Between PD-L2 Expression and the Efficacy of R-CHOP/CHOP Regimen

**Figure 3:**
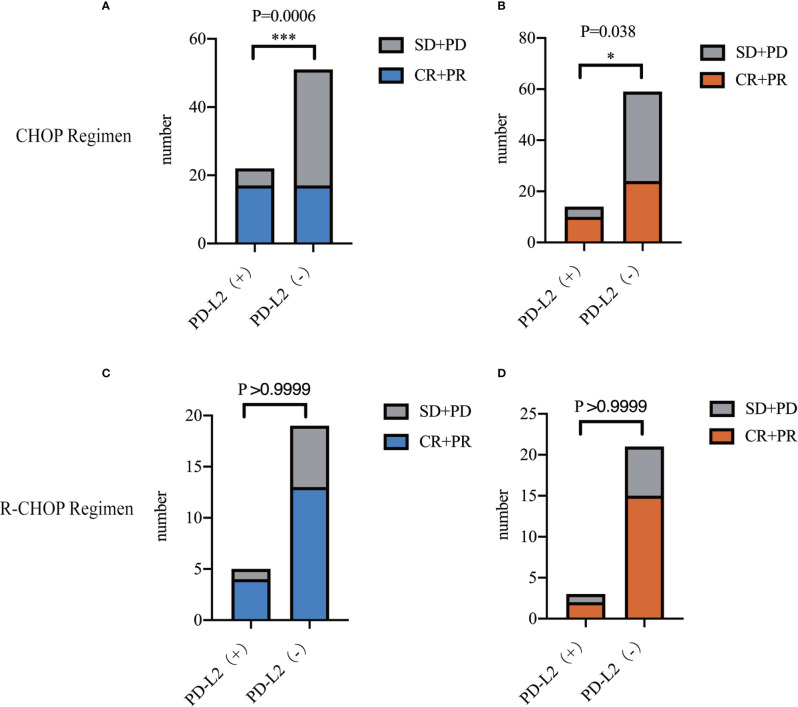
Objective response rates of TC **(A)**/IC **(B)** PD-L2 (+) and PD-L2 (−) patients receiving CHOP regimen. Objective response rates of TC **(C)**/IC **(D)** PD-L2 (+) and PD-L2 (-) patients receiving R-CHOP regimen.

In the 73 patients treated with CHOP regimen, the objective response rate (ORR) was 77.3 and 33.3% in TC PD-L2-positive and -negative patients, respectively. Of the IC PD-L2-positive and -negative patients, the ORR was 71.4 and 40.7%, respectively. The above findings suggested that higher TC and IC PD-L2 expression was associated with higher ORR (**Figures 3A, B**). However, no significant differences were observed between the TC and/or IC PD-L2 expression and the ORR in the 24 patients treated with R-CHOP regimen (*P > 0.05*) (**Figures 3C, D**).

### Association Between PD-L2 Expression and the Immune Infiltration in the TME

**Figure 4:**
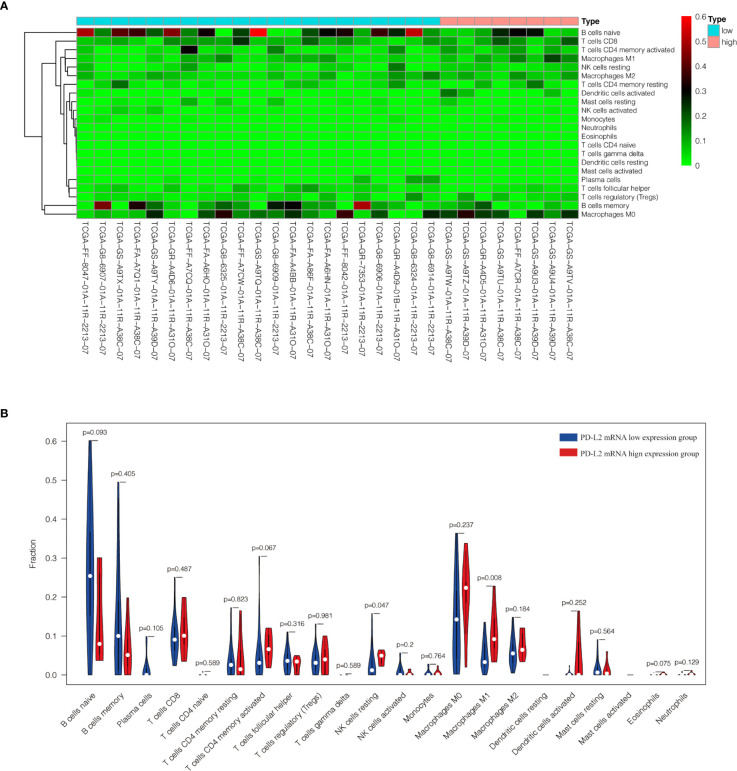
Content of 22 immune cells in 29 DLBCL samples from TCGA database **(A)**. Analysis of the difference of 22 immune cells between high and low expression groups of PD-L2 mRNA **(B)**.

**Figure 5:**
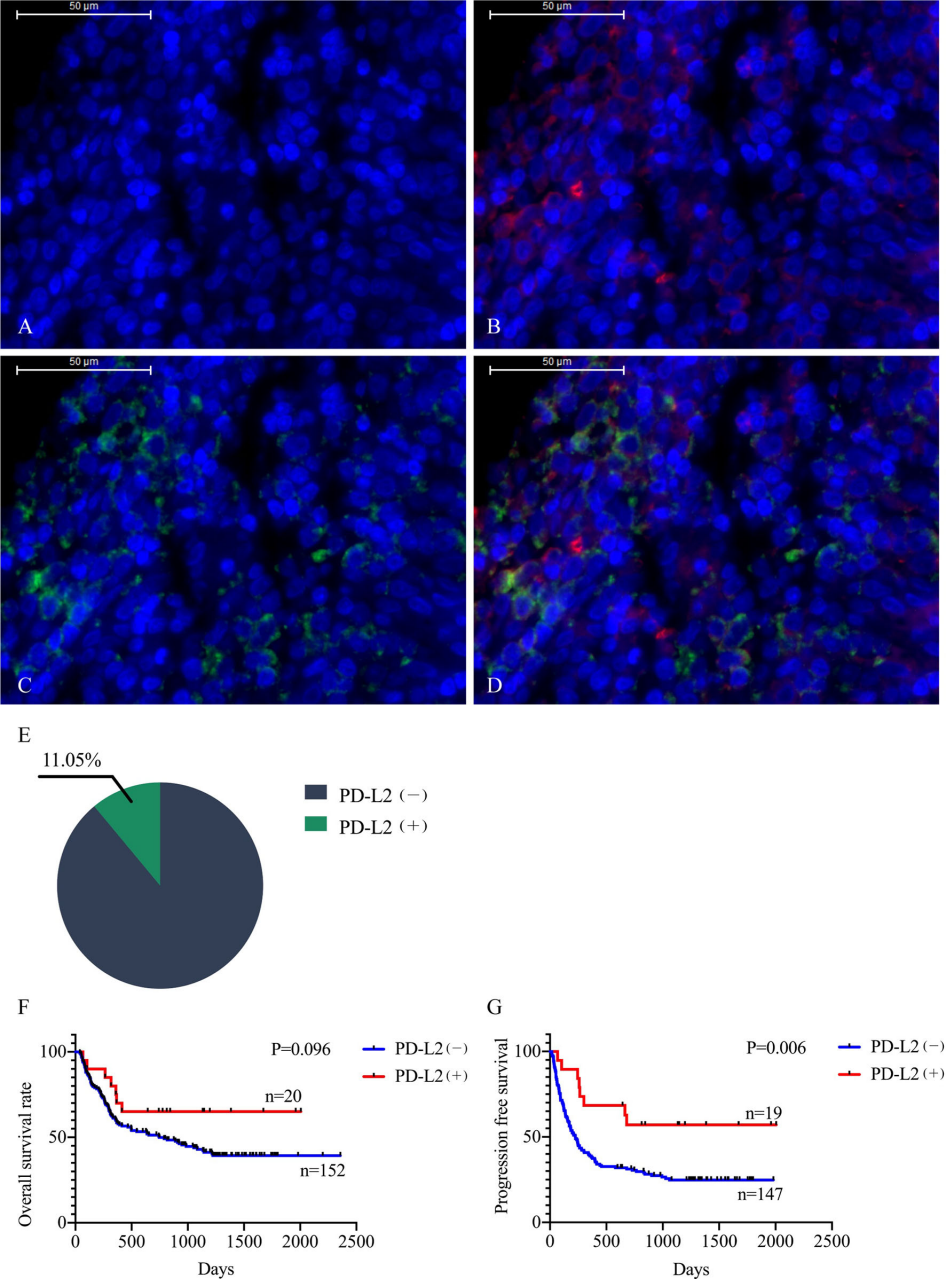
Representative photomicrographs of multiplex immunofluorescence. DAPI: nuclear staining **(A)**, CD68 staining **(B)**, PD-L2 staining **(C)**, Merge (CD68: red signal; PD-L2: green signal) **(D)**. The positive rate of PD-L2 in macrophages **(E)**. Relationship between macrophage PD-L2 (+) and overall survival **(F)** and progression free survival **(G)** curves in newly diagnosed diffuse large B-cell lymphoma.

**Figure 6:**
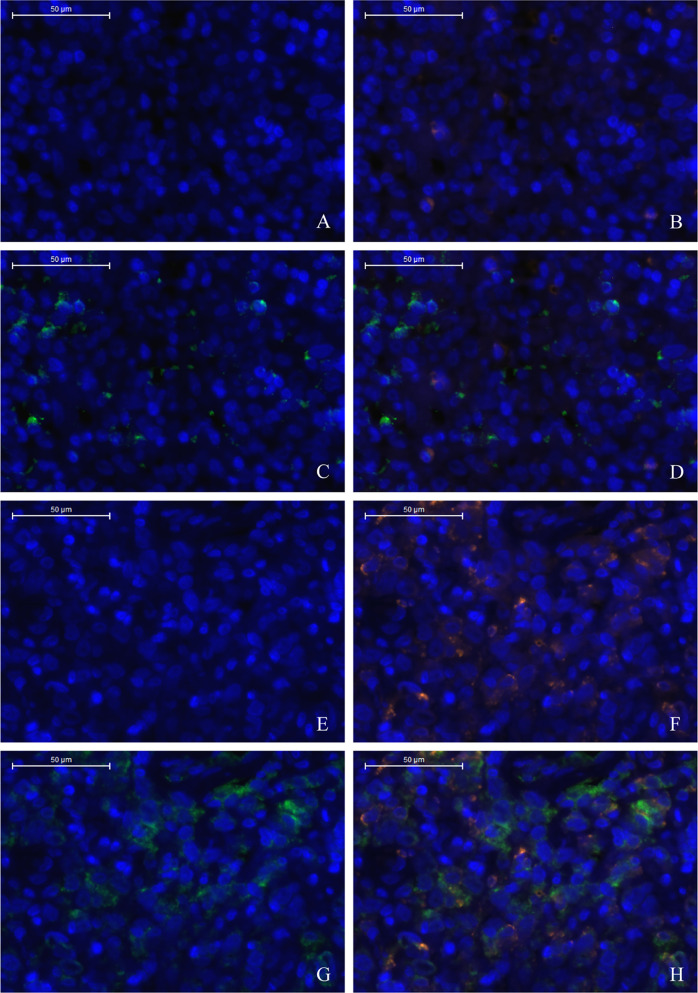
Low density of TC PD-L2 and M1 macrophages **(A–D)**. High density of TC PD-L2 and M1 macrophages **(E–H)**. DAPI: nuclear staining **(A, E)**, CD86 staining **(B, F)**, PD-L2 staining **(C, G)** Merge, **(D, H)**.

**Figure 7:**
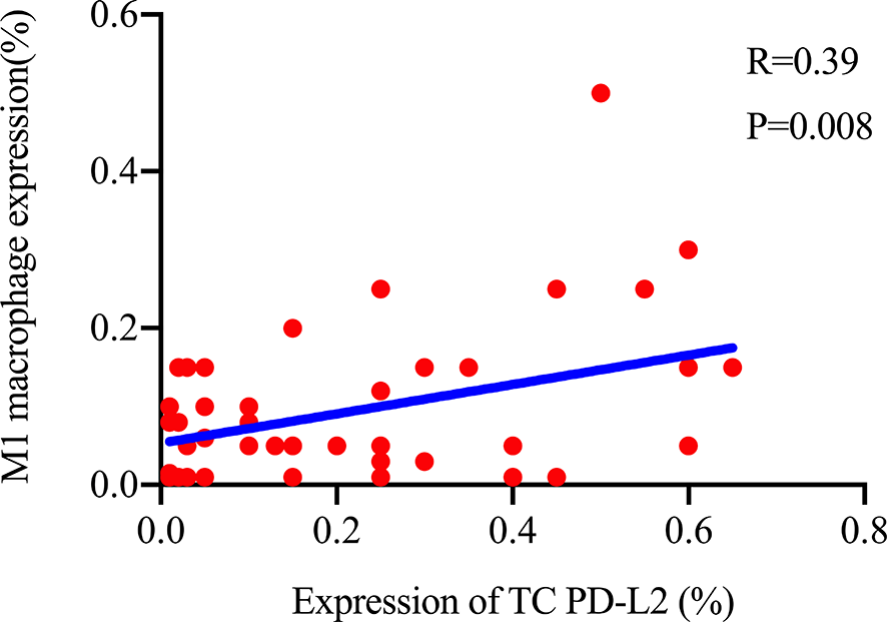
Relationship between the expression of TC PD-L2 and M1 macrophages in tumor tissues.

The TME is mainly composed of tumor infiltrating lymphocytes (TILs), M2 macrophages, M1 macrophages, and myeloid derived suppressor cells (MDSCs), which play an important role in the process of tumor immune escape (28). In lymphomas, studies have shown that PD-L2 is mainly expressed in macrophages (29), and its expression significantly upregulated after treatment with PD-L1 inhibitors (30). PD-L2-positive FL patients usually had a higher percentage of tumor infiltrating immune cells than PD-L2-negative FL patients (23). It has also been shown that increased numbers of tumor-infiltrating immune cells were correlated with improved prognosis in various solid tumor patients. We then asked whether this relationship existed in DLBCL. Firstly, we used CIBERSOFT to analyze immune cells in each DLBCL specimen obtained from the TCGA database (**Figure 4A**). We found that M1 macrophages and dormant NK cells were significantly increased in the PD-L2 high expression group (**Figure 4B**). Subsequently, immunohistochemical staining for CD68, a macrophage surface marker, was carried out on 45 TME PD-L2-positive patients (**Figures 5A–D**). PD-L2 positive macrophages could be found in 20 samples (20/181, 11.05%) (**Figure 5E**). PFS of patients with PD-L2-positive macrophages was significantly longer than for patients without PD-L2-positive macrophages, whereas no significant difference was found in OS (**Figures 5F, G** **)**. Furthermore, multiple fluorescence staining of CD86 was used to evaluate the relationship between PD-L2 expression and M1 macrophage in 45 TME PD-L2-positive patients (**Figure 6**). As expected, the expression level of PD-L2 was positively correlated with the expression status of M1 macrophage markers CD86 (**Figure 7**).

## Discussion

Previous studies have shown that PD-L2 is mainly expressed in primary mediastinal diffuse large B-cell lymphoma (PMBL) and classical Hodgkin lymphoma (cHL) but relatively less in DLBCL (24, 25). In this study, the positive rate of PD-L2 was slightly higher in either the relapsed TCs or ICs. Unlike PD-L1, highly expressed in recurrent cHL, the expression of PD-L2 in recurrent tissues was not significantly higher than that in the newly diagnosed patients, whether in TCs or ICs. Panjwani et al. (24) showed that the positive rate of TC PD-L2 was 6% in American DLBCL patients, and PD-L2 expression was not found in TME. The positive expression of TC PD-L2 was significantly lower than that in this study, and we found that PD-L2 was also expressed on ICs in the TME. The difference of the expression of TC/IC PD-L2 suggests that PD-L2 expression varies among different ethnic populations.

In this study, TC/IC PD-L2 expressed in EBER positive patients was significantly higher than in the negative group. Several studies have confirmed that EBV expression status is significantly correlated with PD-L2 positivity in gastric cancer (31–33). Studies have demonstrated that gastric cancer with EBV (+) and microsatellite instability (MSI) can elicit interferon gamma (IFN-*γ*) driver genes (31), which induce the expression of PD-L1/2 through the signal transducer and activator of transcription (STAT) family (34–36). Meanwhile, TC/IC PD-L2 was associated with ECOG PS score <2, IPI for low risk and intermediate-low risk and Ann Arbor stage I or II. The above mentioned clinical parameters are correlated with better prognosis; this was also validated in the survival analysis. Patients with TC PD-L2 positive had longer OS and PFS compared with negative patients, and patients with high expression of PD-L2 mRNA also had longer OS in two cohorts of DLBCL patients in the GEO database. Xu Monette et al. (37) also found that DLBCL patients with high expression of TC PD-L2 (TPS ≥ 25%) had longer OS by multiplex immunofluorescence staining, and that high TC PD-L2 expression (TPS ≥ 25%) and low PD-1 expression on T cells were all associated with CD80 upregulation. CD80 can interact with CD28 receptors on T cells, reduce the threshold of T cell activation, and promote the survival of T cells (38, 39). Moreover, Shinchi et al. (21) found that patients with high expression of PD-L2 (TPS ≥ 1%) had longer OS and PFS in lung adenocarcinoma. In lung squamous cell carcinoma, Matsubara et al. (22) also pointed out that higher expression of PD-L2 had longer OS than patients with lower expression.

An earlier study showed that PD-L2 was mainly expressed by DCs. DC, as an antigen presenting cell, could induce T cell activation and differentiation through costimulatory signals. PD-L2 expressed on DCs could effectively stimulate the proliferation of T cells (11). Macrophages also share common functions with DCs. In this study, macrophage PD-L2-positive patients had significantly longer PFS. In addition, knockdown of PD-L2 in mice diminished the ability of DCs to activate CD4^+^ T cells, and PD-L2 alone or interacting with CD80 synergistic activation of T cells is independent of the PD-1 axis (40), suggesting that in the initiation of T cell responses, PD-L2 expressed by DCs plays a stimulatory rather than an inhibitory role, and it stimulates T cells through other unknown receptors. Repulsive guidance molecule B (RGMB) has been shown to bind to PD-L2, which can promote T cell expansion (41) and inhibit invasion and metastasis of bladder (42) and breast cancer (43). But it is still unclear about the expression of RGMB in various types of tumors, and when PD-1 and RGMB were expressed simultaneously, the affinity of PD-L2 for both was also unknown. Furthermore, a previous study has shown that high antigen concentration of PD-L2 only reduces the production of cytokines without inhibiting T cell proliferation (14). This also explains why patients with high expression of PD-L2 have longer survival. It indicates that PD-L2, in addition to binding to PD-1 to mediate immunosuppression, also has functions in enhancing the immune response as well as inhibiting the biological behavior of malignant tumors.

In addition, we also found that TC PD-L2 was positively correlated with M1 macrophage specific markers, probably because they had the same regulatory factor, IFN-*γ*. PD-L2 can be upregulated by IFN-*γ* (44), which is also a key factor in inducing macrophages to polarizing M1 macrophages (45). M1 macrophages, a highly efficient antigen-presenting cells, can present antigen to tumor specific CD4^+^ T and CD8^+^ T cells (46), and it also has an anti-tumor effect (47). Similarly, there are other findings suggesting a correlation between PD-L2 expression and ICs in the TME. Tobin et al. (23) found that PD-L2 in FL can effectively reflect the infiltration of immune cells, and patients with high expression of PD-L2 had lower risk of disease progression in 24 months (POD24). Shin et al. (48) revealed that after knocked out of PD-L2, the number of CD8^+^ T cells infiltrated into the tumor as well as their activity decreased in mice. As another ligand of PD-1, PD-L1 is associated with poor prognosis in most cases; it is also found that high expression of PD-L1 in a variety of tumors has a good prognosis (49–53). Patients with high expression of PD-L1 who received radiotherapy usually had a longer survival time (52, 54, 55), and PD-L1 is also associated with the degree of immune cell infiltration (53, 56). These above research results show that PD-L1/2 is associated with better prognosis, and PD-L1/2 induces the infiltration of immune cells into tumors and kills the tumor effectively.

However, other studies have shown that high expression of PD-L2 is associated with poor in esophageal cancer (18), gastric cancer (19) and liver cancer (20). Krittikarux S. et al. (26) have studied the expression of PD-L2 in 88 cases of DLBCL who received R-CHOP regimen using immunohistochemistry, with 5% as the cut-off value, and found that the TC PD-L2 positive rate was 68.4%, which was significantly higher than that in the present study. Although shorter survival was observed in patients with higher PD-L2 expression compared with those with lower expression, no statistical significance was found. The results of the study conflict with our study, which may be the use of different antibodies and cut-off values. In addition, rituximab was included in the chemotherapy regimens in the study of Krittikarux S, while most of the patients in this study were treated with the CHOP regimen. Different treatment options will also lead to different results.

By analyzing response rates to chemotherapy, we found that patients in the TC PD-L2-positive (TPS ≥ 1%) and IC PD-L2-positive (IPS ≥ 1%) groups had significantly higher ORR rates to receive six cycles of CHOP compared with the negative group, and that patients in the TC or IC PD-L2-positive group had ORR rates very close to those of the R-CHOP regimen. Considering that TC PD-L2 positive is an independent prognostic factor of PFS, PD-L2 is likely to become a molecular marker for disease progression in DLBCL. However, due to the low positive rate of PD-L2, whether it can effectively predict the clinical efficacy of CHOP regimen remains to be further verified by a larger population.

Prednisone, which is included in the CHOP regimen, is one of the currently frequently used glucocorticoids and has immunosuppressive effects, playing a key physiological role in the feedback inhibition of inflammatory responses and immune system homeostasis. But it is still unclear whether it will affect the binding of PD-1 and PD-L1/2. A recent study has observed that in the 640 patients receiving PD-1/PD-L1 inhibitor treatment, the ORR rate of patients receiving 10 mg/d prednisone decreased, and PFS and OS significantly shortened; in a further multivariate analysis, prednisone was significantly associated with a decrease in progression free survival (57). It can be seen that prednisone can reduce the clinical benefits of immune checkpoint inhibitors. One reason may be that the immunosuppressive effect of prednisone is higher than that of the PD-1 axis. Therefore, the immunosuppression of prednisone makes the immune cells unable to exert tumor killing effect. Secondly, prednisone may directly affect the binding or dissociation between PD-1 and ligands, which still needs further verification. However, when prednisone is included in the CHOP/R-CHOP regimen, the survival of DLBCL is prolonged due to the specificity of the origin of hematological malignancies. So, we suspect that prednisone may conceal the immunosuppression induced by the PD-1 axis, taking into account the inhibitory effect of PD-L2, so the TC/IC PD-L2 positive group has a higher clinical benefit rate.

In general, we found that PD-L2 was expressed on both TCs and ICs in DLBCL by immunohistochemistry, patients with PD-L2-positive on TCs had significantly longer PFS and OS, and patients with PD-L2-positive on TCs and TME who received the CHOP regimen resulted in higher ORR. Besides participating in immunosuppression, we also observed that PFS in macrophages positive for PD-L2 was significantly longer than that in negative ones. PD-L2 may induce M1 macrophages to infiltrate into tumor tissues and mediate antitumor effects, which may play a role in inhibiting DLBCL. However, when PD-L2 is expressed differently in various types of tumors, its prognostic effects may be distinct under different treatment strategies. Furthermore, its related mechanism is still unclear, which needs further study.

## Data Availability Statement

The raw data supporting the conclusions of this article will be made available by the authors without undue reservation.

## Ethics Statement

Written informed consent was obtained from the individual(s) for the publication of any potentially identifiable images or data included in this article.

## Author Contributions

QG and JL have contributed equally to this work. All authors contributed to the article and approved the submitted version.

## Funding

This work was supported by grants from the National Natural Science Foundation of China (81700189), Nantong Science and Technology Project (MS12019017, HS2019003), and Nantong Health Commission Project (MB2019022).

## Conflict of Interest

The authors declare that the research was conducted in the absence of any commercial or financial relationships that could be construed as a potential conflict of interest.
